# Iatrogenic acute aortic dissection caused by intervention for spontaneous coronary artery dissection: a surgical case report

**DOI:** 10.1186/s13019-020-01333-6

**Published:** 2020-09-29

**Authors:** Yuki Hayashi, Makoto Taoka, Shunji Osaka, Satoshi Unosawa, Masashi Tanaka

**Affiliations:** Department of Cardiovascular Surgery, Nihon University School of Medicine, 30-1 Oyaguchi-kamicho, Itabashi-ku, Tokyo, 173-8610 Japan

**Keywords:** Cardiovascular surgery, Spontaneous coronary artery dissection, Iatrogenic acute aortic dissection

## Abstract

**Background:**

Iatrogenic acute aortic dissection (AAD) caused by cardiovascular intervention is rare. Also rare is spontaneous coronary artery dissection (SCAD), a form of acute coronary syndrome, which develops in relatively young women without coronary risk factors. We encountered type A iatrogenic AAD caused by an intervention for SCAD.

**Case presentation:**

A 53-year-old woman was brought to our hospital after cardiopulmonary resuscitation. She was diagnosed with acute coronary syndrome caused by SCAD, and percutaneous coronary intervention was carried out on her distal left anterior descending artery. The dissection proceeded to the proximal left anterior descending artery and left main coronary artery trunk, so additional percutaneous coronary intervention was performed on the left circumflex artery. After the intervention, type A AAD occurred with a primary entry tear from the left main coronary artery trunk, and computed tomography showed a type A AAD of the aortic arch. We performed emergency ascending aorta replacement and coronary artery bypass grafting to the left anterior descending artery and left circumflex artery. The patient had an uneventful recovery after the operation and was discharged on postoperative day 25.

**Conclusion:**

To our knowledge, this is the first report of an iatrogenic AAD caused by percutaneous coronary intervention for SCAD.

## Background

With advancements in cardiovascular interventional techniques and materials, the number of percutaneous coronary intervention (PCI) procedures is increasing, as is the number of patients who receive complex and higher-risk PCIs. Iatrogenic acute aortic dissection (IAAD) is one of the complications of coronary angiography and PCI. It can be fatal, but is very rare, with an incidence of 0.01–0.04% after PCI [1]. IAAD during coronary intervention can be fatal, and a prompt aortic repair is mandatory for saving a life.

Spontaneous coronary artery dissection (SCAD) is a rare form of acute coronary syndrome (ACS) and one of the causes of sudden cardiac death. It particularly affects young women without coronary risk factors, often those aged 40–50 years or who are pregnant [2, 3]. Most cases of SCAD are detected by coronary angiography, intravascular ultrasound and optical coherence tomography, which show an intimal flap in the coronary artery. The incidence rate of SCAD is 0.07–1.1% in coronary angiography [2, 3]. Hormonal changes during pregnancy, fibromuscular dysplasia, intensive exercise and connective tissue diseases (Marfan syndrome, Ehlers–Danlos syndromes) are associated with SCAD [2]. Patients with SCAD who have acute or ongoing myocardial ischemia are treated with PCI, whereas those who are stable or asymptomatic are observed without revascularization. The recurrence rate of SCAD is high (29.4% in 10 years, and 47.4% for all cardiovascular events) [4]. In most cases, the dissection is localized to the coronary artery. We report herein the rare case of type A IAAD caused by coronary intervention for SCAD.

## Case presentation

**Fig. 1:**
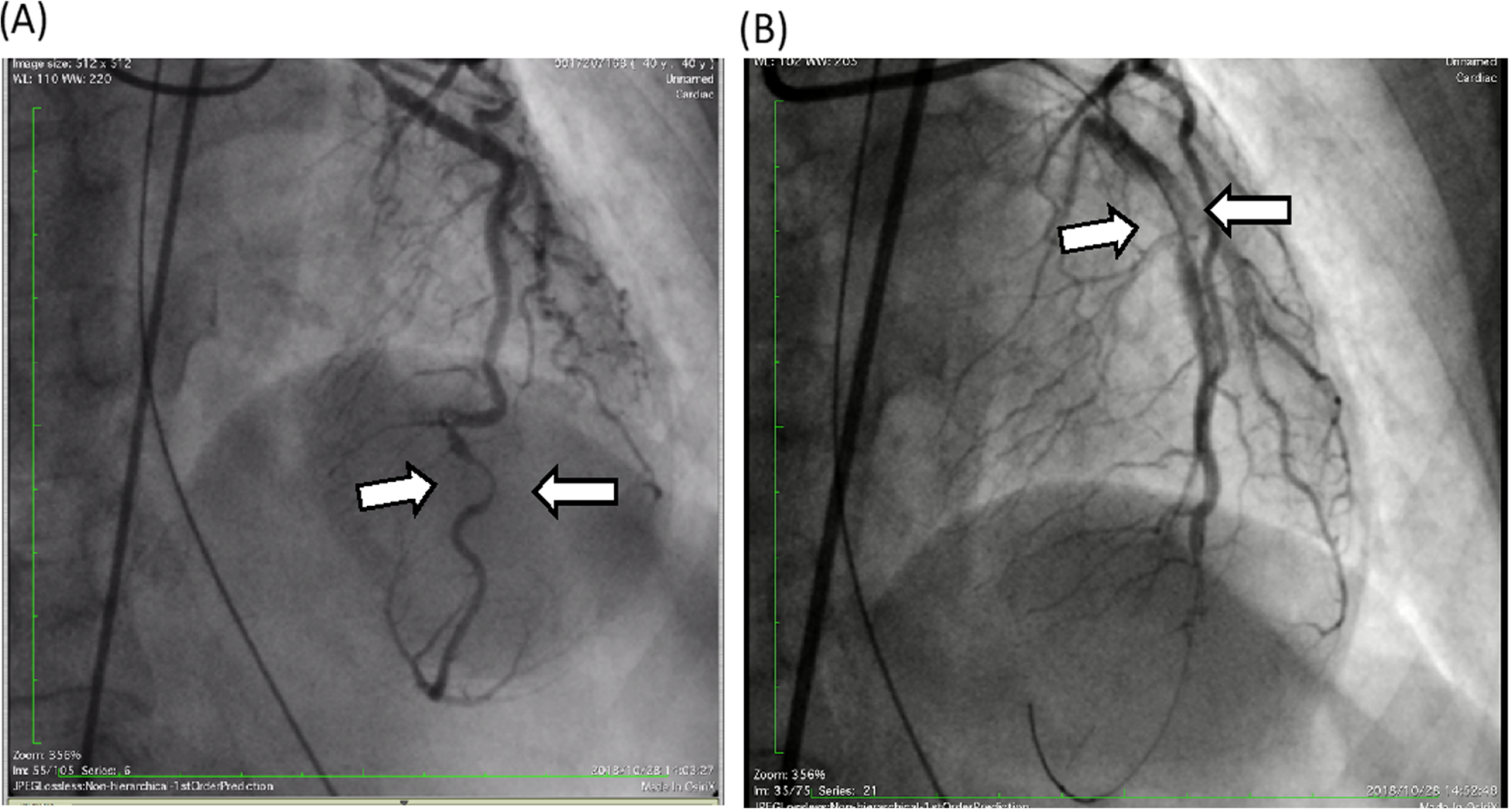
Coronary angiography of dissected artery. **a**, Dissection of distal left anterior descending artery (LAD; arrows). **b**, After percutaneous coronary intervention to the distal LAD, the proximal LAD was found to be newly dissected (arrows)

**Fig. 2:**
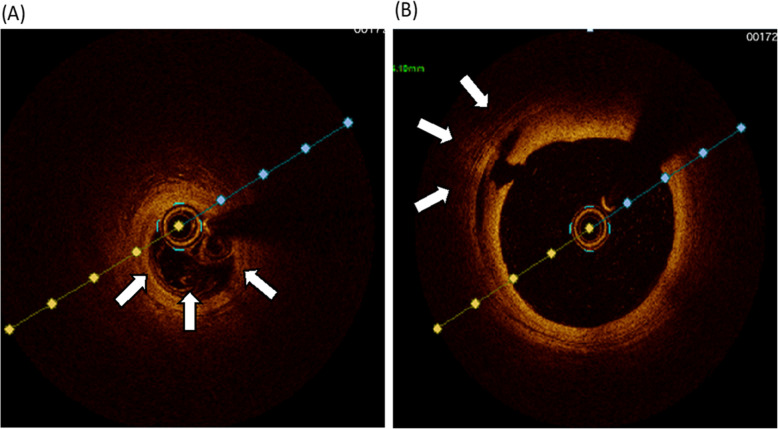
Optical coherence tomography of dissected artery. **a**, Distal left anterior descending artery (LAD) dissection (arrows). **b**, Proximal LAD similarly dissected

**Fig. 3:**
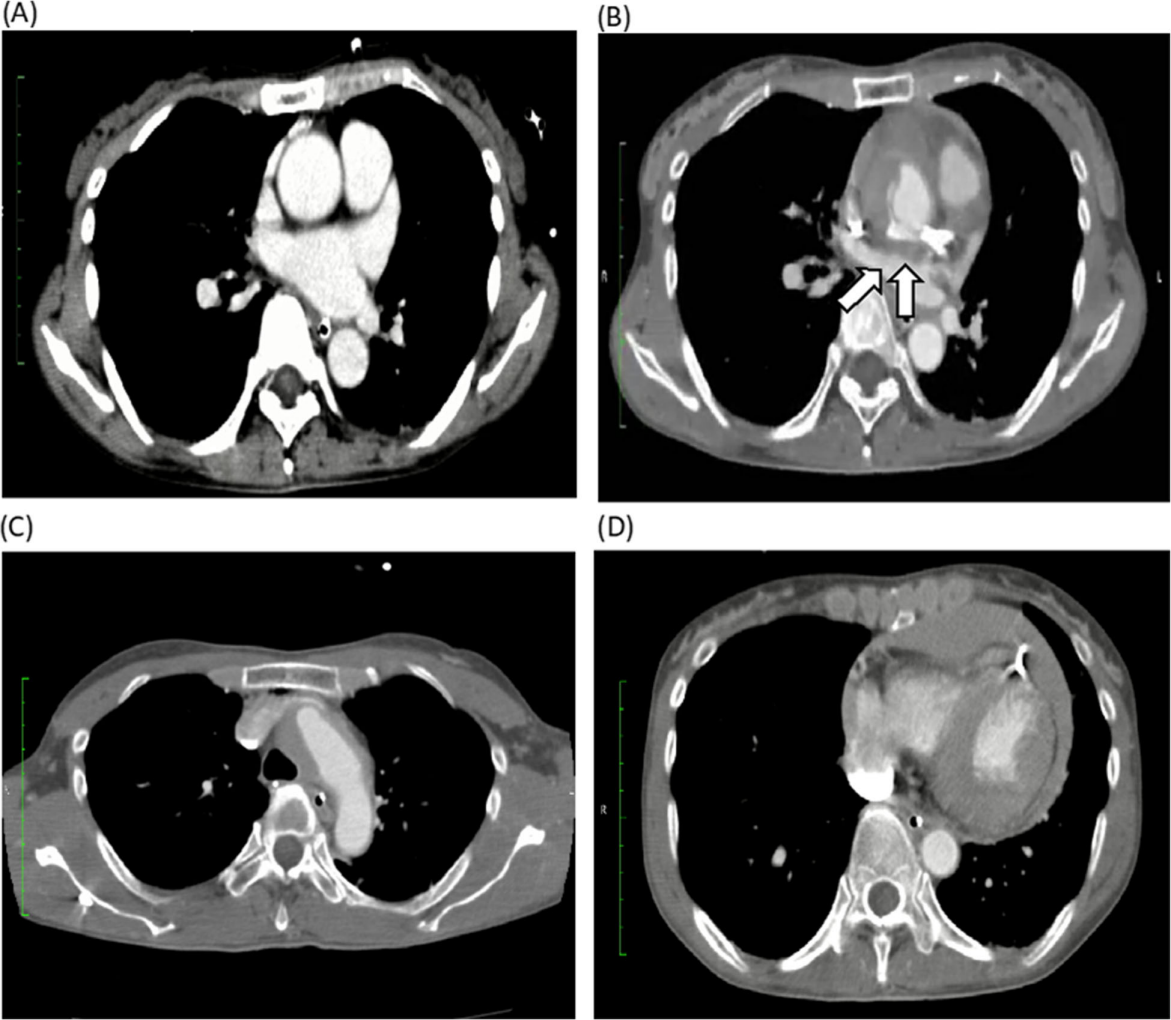
Computed tomography of the patient’s chest. **a**, Before percutanous coronary intervention (PCI), no aortic dissection was observed. **b**, After PCI, a new dissection appeared in the ascending aorta (arrow). The false lumen is non-communicating. **c**, Dissected aortic arch. **d**, A massive bloody pericardial fluid appeared, which was not present before PCI

A 53-year-old nulliparous woman was brought to our hospital after resuscitation from cardiopulmonary arrest. She was found lying in the park and a rescue team attempted defibrillation. On admission, an electrocardiogram showed V4–6 ST elevation, transthoracic echocardiography showed apical wall hypokinesis, and a computed tomography (CT) scan showed no aortic dissection or pericardial effusion. Creatine kinase level was 149 U/L (normal range, 0–165), creatine kinase myocardial band level was 20 U/L (normal range 0.2–5.0), and cardiac troponin I level was 0.26 ng/ml (normal range, 0.01–0.05). Because myocardial infarction was suspected, we performed emergency coronary angiography. The distal left anterior descending artery (LAD) was dissected, and PCI was performed. After this procedure, the proximal LAD and left circumflex artery were found to be newly dissected, as confirmed by coronary angiography (Fig. 1) and optical coherence tomography (Fig. 2), so PCI was also carried out in these arteries. Pericardial effusion collection was recognized by transthoracic echocardiography and increased gradually, and CT showed type A AAD with a primary entry tear from the left main coronary artery trunk (LMT), which had not seen when the patient was arrived at our hospital (Fig. 3). The patient’s systolic blood pressure decreased to 60 mmHg, so we proceeded with surgery.

**Fig. 4:**
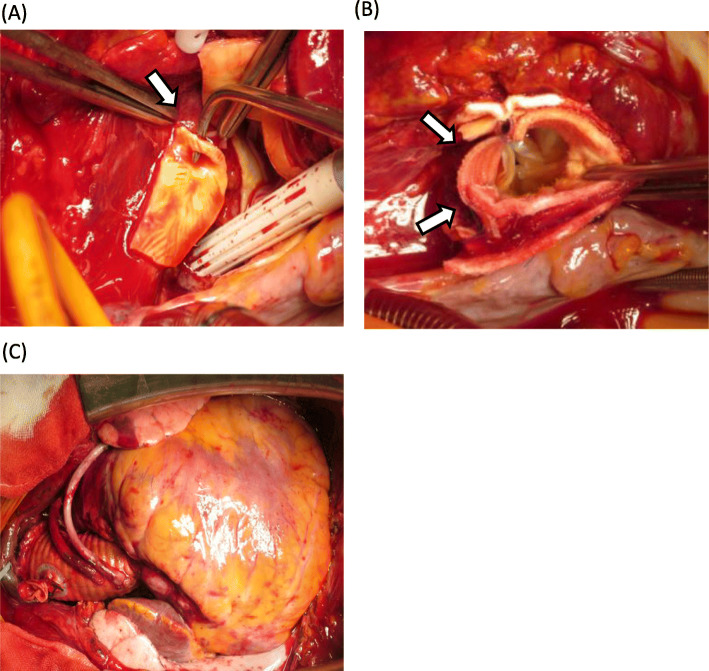
Intraoperative photographs. The left side of the images are cranial. **a**, The forceps tip is in the coronary artery medial toward the left main coronary artery trunk ostium, which is the primary entry point (arrow). **b**, Arrows point to the ascending aorta repaired by trapezoid artificial vascular graft. **c**, Coronary artery bypass graft of the left anterior descending artery and left circumflex artery by great saphenous vein, and ascending aortic replacement by artificial vascular graft

We performed a median sternotomy under general anesthesia. Bloody pericardial fluid and a hematoma in the fatty tissue of the right ventricle were present. Cardiopulmonary bypass was established with left femoral artery cannulation and bicaval venous cannulation, then the body temperature was lowered. When the bladder temperature reached 26 °C, circulatory arrest was initiated and the ascending aorta was opened. No hematoma was observed in the false lumen or primary entry tear. We hollowed out the left coronary ostium from the ascending aorta and observed it carefully. We found the primary entry, which is the posterior wall of the LMT ostium, and the outer membrane of the LMT was blown-out (Fig. 4a). The distal end of the ascending aorta was sutured to a 24-mm artificial vascular graft, and antegrade blood delivery was started from the side branch of the vascular graft. Because the primary entry was at the LMT, it was impossible to perform ostial patch plasty, so we closed the LMT ostium by continuous suture and bypassed grafting to LAD and left circumflex artery. The hole in the ascending aorta (the LMT ostium) was closed using a trapezoid artificial vascular graft patch (Fig. 4b). The proximal ascending aorta was sutured to the artificial vascular patch and the clamp was removed from the ascending aorta. As the bladder was warming, we carried out a coronary artery bypass graft (CABG) to the LAD and left circumflex artery using great saphenous vein grafts (Fig. 4c).

Weaning from the cardiopulmonary bypass went smoothly. Bypass time was 192 min, circulatory arrest time was 17 min, cross-clamp time was 156 min, and operation time was 321 min. Postoperative transthoracic echocardiography revealed anteroseptal hypokinesis with an ejection fraction of 54%. The postoperative course was uneventful and the patient was discharged from our hospital on postoperative day 24. However, on postoperative day 64, she was brought back to our hospital with cardiopulmonary arrest, but did not recover. The cause of death was not identified, because of her family did not expect autopsy.

## Discussion and conclusions

IAAD is a rare complication during coronary angiography, PCI or cardiac surgery. Its incidence during cardiac catheterization is 0.01–0.02% and during cardiac surgery is 0.06–0.23% [5]. There are no specific guidelines for IAAD management, but surgery must be performed quickly if the patient’s clinical condition deteriorates or does not respond sufficiently to other interventions such as pericardiocentesis.

There have been few reports of IAAD since the first case reported by Matar et al. in the 1960s [6]. Bartosz et al. published the clinical features and 30-day outcomes of spontaneous AAD or IAAD in 50 German centers [5], reporting the incidence of IAAD 0.01–0.02% during cardiac catheterization, and that coronary malperfusion and additional CABG were more frequently needed in IAAD than spontaneous AAD. Both groups showed a 30-day mortality of about 16%. Verevkin et al. [1] reported data from patients who underwent emergency cardiac surgery for post-PCI iatrogenic lesions including dissection, occlusion or perforation of the coronary artery. The 30-day mortality of these procedures was 20.8%, and the long-term survival of patients who lived past 30 days was 89.83, 79.04, 64.06, and 60.06% at 1, 5, 10, and 12 years, respectively. These studies indicate that the mortality rate of IAAD is high, and preoperative conditions are strongly associated with mortality in the perioperative to late-postoperative periods.

SCAD is an infrequent form of ACS and affects healthy young women at low risk of atherosclerosis. Of all SCAD cases, 75% are in women and one-third of these occur during pregnancy. Most cases of SCAD are diagnosed by coronary angiography, with an incidence of 0.07–1.1% all of coronary angiographies. Moreover, SCAD recurs in 13–22% of cases [7]. Patients who have progression of myocardial ischemia are treated with aggressive therapy (PCI or surgery). Tweet et al. reported that of 87 patients initially treated for SCAD, 39 underwent PCI, 31 required no revascularization, 13 were treated with fibrinolytics, and only 4 required CABG; of the PCI procedures, 15 were complicated by technical failure and 5 were changed to CABG [4]. Saw et al. reported that 83.2% of patients with SCAD were treated conservatively, 16.5% underwent PCI, and 2.1% underwent CABG. Additionally, the 10-year rate of major adverse cardiac events, recurrent SCAD, myocardial infarction, congestive heart failure or death was 47.4% [2]. Nakanishi et al. also reported that patients with SCAD had a much higher incidence of major adverse cardiac events than those without SCAD [7]. Several reports conclude that PCI for SCAD does not result in favorable outcomes, and SCAD patients have a high risk of recurrence within 30 days of developing the first coronary dissection [2, 4]. If the anatomy is unsuitable, or in cases of dissection involving the left main coronary artery, CABG should be considered [8]. When performing CABG for SCAD, grafts more distal than the dissected vessel must be sutured or, if that is not possible, the intimal tear must be repaired [7].

It is very rare that dissection progresses to the aortic arch from SCAD. Almost all cases of SCAD requiring surgery undergo CABG, likely because the dissection does not reach the ascending aorta. One case was reported by Ridvan et al. [9] in a 61-year-old woman with SCAD of the distal right coronary artery that extended to the sinus of Valsalva, caused by injection of contrast agent. Primary repair of the aortic dissection was carried out.

In summary, we encountered a rare case of type A AAD, in which the dissection caused by SCAD progressed to the aortic arch due to PCI. The etiology of SCAD is not known, but its recurrence rate is higher than in other ACSs, and it affects younger patients [2, 3, 8]. Therefore, treatment should be chosen carefully, and patients should be followed-up frequently. The mortality rate of IAAD is also high. How best to approach SCAD remains a matter of debate; however, it is clear that surgery must be performed diligently to avoid exacerbating the patient’s condition. IAAD and SCAD are rare, and therefore, we report this case to improve the management and care of patients with these conditions.

## Data Availability

All data generated or analyzed during this study are included in this published article and its supplementary information files.

## Abbreviations

AAD: Acute aortic dissection
ACS: Acute coronary syndrome
CABG: Coronary artery bypass grafting
CT: Computed tomography
IAAD: Iatrogenic acute aortic dissection
LAD: Left anterior descending artery
LMT: Left main coronary artery trunk
PCI: Percutaneous coronary intervention
SCAD: Spontaneous coronary artery dissection

## References

[1] Verevkin A, von Aspern K, Leontyev S, Lehmann S, Borger MA, Davierwala PM. Early and long-term outcomes in patients undergoing cardiac surgery following iatrogenic injuries during percutaneous coronary intervention. J Am Heart Assoc. 2019;8:e010940. 10.1161/JAHA.118.010940 PMC6405713 30612504

[2] Jacqueline S, Karin H, Eve A, Tara S, Roshan P, Andrew S (2017). Spontaneous coronary artery dissection. J Am Coll Cardiol.

[3] Alfonso F (2012). Spontaneous coronary artery dissection: new insights from the tip of the iceberg?. Circulation.

[4] Tweet MS, Hayes SN, Pitta SR, Simari RD, Lerman A, Lennon RJ (2012). Clinical features, management, and prognosis of spontaneous coronary artery dissection. Circulation.

[5] Rylski B, Hoffmann I, Beyersdorf F, Suedkamp M, Siepe M, Nitsch B (2013). Iatrogenic acute aortic dissection type a: insight from the German registry for acute aortic dissection type a (GERAADA)†. Eur J Cardiothorac Surg.

[6] Matar AF, Ross DN (1967). Traumatic arterial dissection in open-heart surgery. Thorax..

[7] Nakashima T, Noguchi T, Haruta S, Yamamoto Y, Oshima S, Nakao K (2016). Prognostic impact of spontaneous coronary artery dissection in young female patients with acute myocardial infarction: a report from the angina pectoris–myocardial infarction multicenter investigators in Japan. Int J Cardiol.

[8] Amelia Y, Jacqueline S (2015). Spontaneous coronary artery dissection—A review. Cardiovasc Diagn Ther.

[9] Yalçin R, Taçoy GA, Timurkaynak T, Cengel A (2003). Dissection of the Aortic Sinus of Valsalva During Coronary Angiography in a Patient with Spontaneous Coronary Artery Dissection. Anatol J Cardiol.

